# FHL1C induces apoptosis in notch1-dependent T-ALL cells through an interaction with RBP-J

**DOI:** 10.1186/1471-2407-14-463

**Published:** 2014-06-22

**Authors:** Wei Fu, Kai Wang, Jun-Long Zhao, Heng-Chao Yu, San-Zhong Li, Yan Lin, Liang Liang, Si-Yong Huang, Ying-Min Liang, Hua Han, Hong-Yan Qin

**Affiliations:** 1 Department of Hematology, Tangdu Hospital, Fourth Military Medical University, Xi’an 710038, People’s Republic of China; 2 State Key Laboratory of Cancer Biology, Department of Medical Genetics and Developmental Biology, Fourth Military Medical University, Xi’an 710032, People’s Republic of China

**Keywords:** T-cell acute lymphoblastic leukemia, Notch signaling, FHL1C, RBP-J, Apoptosis

## Abstract

**Background:**

Aberrantly activated Notch signaling has been found in more than 50% of patients with T-cell acute lymphoblastic leukemia (T-ALL). Current strategies that employ γ-secretase inhibitors (GSIs) to target Notch activation have not been successful. Many limitations, such as non-Notch specificity, dose-limiting gastrointestinal toxicity and GSI resistance, have prompted an urgent need for more effective Notch signaling inhibitors for T-ALL treatment. Human four-and-a-half LIM domain protein 1C (FHL1C) (KyoT2 in mice) has been demonstrated to suppress Notch activation in vitro, suggesting that FHL1C may be new candidate target in T-ALL therapy. However, the role of FHL1C in T-ALL cells remained unclear.

**Methods:**

Using RT-PCR, we amplified full-length human FHL1C, and constructed full-length and various truncated forms of FHL1C. Using cell transfection, flow cytometry, transmission electron microscope, real-time RT-PCR, and Western blotting, we found that overexpression of FHL1C induced apoptosis of Jurkat cells. By using a reporter assay and Annexin-V staining, the minimal functional sequence of FHL1C inhibiting RBP-J-mediated Notch transactivation and inducing cell apoptosis was identified. Using real-time PCR and Western blotting, we explored the possible molecular mechanism of FHL1C-induced apoptosis. All data were statistically analyzed with the SPSS version 12.0 software.

**Results:**

In Jurkat cells derived from a Notch1-associated T-ALL cell line insensitive to GSI treatment, we observed that overexpression of FHL1C, which is down-regulated in T-ALL patients, strongly induced apoptosis. Furthermore, we verified that FHL1C-induced apoptosis depended on the RBP-J-binding motif at the C-terminus of FHL1C. Using various truncated forms of FHL1C, we found that the RBP-J-binding motif of FHL1C had almost the same effect as full-length FHL1C on the induction of apoptosis, suggesting that the minimal functional sequence in the RBP-J-binding motif of FHL1C might be a new drug candidate for T-ALL treatment. We also explored the molecular mechanism of FHL1C overexpression-induced apoptosis, which suppressed downstream target genes such as Hes1 and c-Myc and key signaling pathways such as PI3K/AKT and NF-κB of Notch signaling involved in T-ALL progression.

**Conclusions:**

Our study has revealed that FHL1C overexpression induces Jurkat cell apoptosis. This finding may provide new insights in designing new Notch inhibitors based on FHL1C to treat T-ALL.

## Background

T-cell acute lymphoblastic leukemia (T-ALL) is an aggressive neoplasm that originates from immature T-cells. Although the currently used multi-agents chemotherapy results in 5-year relapse-free survival rates of over 75% in children and over 50% in adults, relapse usually is associated with resistances against chemotherapy and a very poor prognosis [1-3]. Therefore, it is essential to elucidate the molecular mechanisms underlying T-ALL progression to discover new therapeutic targets for the treatment of T-ALL.

Mutations in the Notch1 receptor have been demonstrated as the etiological cause of T-ALL [4,5]. The first evidence of oncogenic Notch signaling was observed in T-ALL patients, involving translocation of a portion of the human Notch1 gene to the TCR locus [6]. However, this event is rare in human T-ALL (less than 1%). In fact, more than 50% of T-ALL patients carry Notch1-activating mutations that are usually in the heterodimerization (HD) domain and proline/glutamic acid/serine/threonine-rich motifs (PEST) of the Notch1 receptor, which result in delayed degradation of Notch1 [7]. Notch1 is one of the four mammalian Notch receptors that are single-pass transmembrane proteins consisting of functional extracellular, transmembrane, and intracellular domains. When the Notch receptor is triggered upon interaction with its ligands on neighboring cells, the Notch intracellular domain (NIC) is released from the membrane after proteolytic cleavages executed by γ-secretase-containing protease complexes. The NIC enters the nucleus and associates with the DNA-binding transcription factor RBP-J through its N-terminal RAM (RBP-J association molecule) domain, which transactivates promoters harboring RBP-J-binding sites by dissociating co-repressors, such as SMRT/N-CoR, HDAC, and MINT [1,8], and recruiting co-activators including Mastermind-like (MAML) and p300/CBP [9]. In T-ALL, activated Notch1 regulates cell proliferation and apoptosis by modulating the level and activities of the related molecules/pathways such as Hes1, c-Myc, PI3K/AKT, and NF-κB through canonical (RBP-J-dependent) and/or non-canonical (RBP-J-independent) signals [10,11].

Considering the critical role of Notch activation in the progression of T-ALL, efforts have been made to cure T-ALL by blocking Notch signaling. Small molecule γ-secretase inhibitors (GSIs), which block the critical proteolytic steps required for Notch activation, can be applied for T-ALL treatment, but the clinical outcomes have been unsatisfactory. These outcomes might be attributed to the fact that γ-secretase is not specific for Notch receptors, and more importantly, GSIs only affect ligand-dependent Notch activation, not ligand-independent Notch activation resulting from chromosome translocation or point mutations. In addition, gastrointestinal toxicity and weak anti-leukemic effects on T-ALL also hinder the clinical application of GSIs [12,13]. Another target for blocking Notch signaling in malignant T cell leukemia is RBP-J that mediates the effects of Notch1 mutants on downstream gene expression. Expression of a dominant-negative MAML1 (DN-MAML1) in T-ALL cell lines has been shown to antagonize Notch1 activation [14,15]. Subsequently, Moellering et al. designed a stable α-helical peptide derived from MAML1 (SAHM1) based on the structure of DN-MAML1. They found that SAHM1 directly impedes assembly of the Notch1 transactivation complex in the nucleus and reduces malignant cell proliferation and promotes apoptosis. In contrast to GSIs, DN-MAML1 and SAHM1 inhibit Notch activation more efficiently because of their direct inhibition of Notch signals at the transcriptional factor level. However, as a multifunctional transcription activator, MAML1 is also not specific for Notch signaling [16]. Thus, more effective Notch signal inhibitors are still required for the treatment of T-ALL.

Human four-and-a-half LIM domain protein 1C (FHL1C) (KyoT2 in mice) belongs to the four-and-a-half LIM domain protein family and is an alternatively spliced form of FHL1A/KyoT1. Selective use of exons results in a frame shift in translation, generating a WW-containing motif at the C-terminus of FHL1C, which can bind to RBP-J. Without a transcription activation domain, FHL1C/KyoT2 has been demonstrated to compete with NIC for RBP-J binding and suppress RBP-J-mediated Notch activation in vitro [8]. These findings suggest that FHL1C may be another therapeutic target of T-ALL, but the role of FHL1C remains to be investigated in T-ALL cells. In the present study, we addressed this issue using T-ALL clinical samples and the T-ALL cell line Jurkat. We found that the expression level of FHL1C was lower in the peripheral blood mononuclear cells (PBMCs) of T-ALL patients than that in the controls. Overexpression of FHL1C or its various truncates containing the RBP-J-binding site or the minimal RBP-J-binding motif, all resulted in Jurkat cell apoptosis. Mechanistically, FHL1C-induced Jurkat cell apoptosis involved suppression of downstream target genes and key pathways of Notch signaling in T-ALL, including PI3K-AKT and NF-κB. These findings shed light on the design of new Notch inhibitors based on FHL1C to treat T-ALL.

## Methods

### Vector construction

Total RNA was extracted from a human skeletal muscle biopsy and then reverse transcribed using a commercially available kit from TAKARA (Dalian, China) with an oligo-dT primer. This patient had signed informed consent, and the protocol involving human samples was approved by the Ethics Committee of Tangdu Hospital, Fourth Military Medical University. FHL1C (GeneBank accession number: AF220153.1) was amplified by PCR with specific primers (Forward primer, 5′-ATGGCGGAGAAGTTTGACTGCCACTACT-3′; Reverse primer, 5′-TCACGGAGCATTTTTTGCAGTGGAAGCA-3′) (Additional file 1: Table S1). The 585 bp PCR product was cloned and confirmed by DNA sequencing. The full-length FHL1C cDNA was inserted into the expression vectors pEGFP-C1 (Clontech, Mountain View, CA) and pCMV-Myc (Clontech) to generate pEGFP-FHL1C and pCMV-Myc-FHL1C, respectively.

To construct EGFP-tagged truncates of FHL1C, LIM1, LIM2, and the C-terminal RBP-J-binding motif (RBP^motif^) of FHL1C, various fragments were subcloned by PCR with the primers listed in Additional file 1: Table S1, and pEGFP-FHL1C expression vector was used as the template. The LIM1 and LIM2 domains were fused in frame at the 3′ terminus to the RBP^motif^ to generate LIM1R and LIM2R, respectively. LIM1R, LIM2R, and RBP^motif^ were then inserted in frame into pEGFP-C1 to generate pEGFP-LIM1R, pEGFP-LIM2R, and pEGFP-RBP^motif^ (Additional file 2: Figure S3A). To construct vectors for expression of EGFP fused to the minimal RBP^motif^ of FHL1C, double-stranded oligonucleotides encoding VWWPM, PVWWPMK, and APVWWPMKD peptides were synthesized and cloned in frame downstream of EGFP in pEGFP-C1. The plasmids were confirmed by DNA sequencing.

### Patients, RNA extraction, RT-PCR, Sequencing

Blood samples were collected from T-ALL patients and normal healthy individuals (Additional file 3: Tables S3 and Additional file 4: Table S4). All patients and normal individuals involved in the study had signed informed consents for the use of their blood samples, except for children under the age of 18, who had their informed consents signed by their parents as their representatives. The protocols involving human samples were approved by the Ethics Committee of Tangdu Hospital, Fourth Military Medical University. Diagnoses had been made according to standard morphological, immunological, and molecular genetics criteria. PBMCs were separated by Ficoll-Hypaque density gradient centrifugation. Total RNA was extracted from PBMCs and Jurkat cells using Trizol reagent (Invitrogen, Carlsbad, CA), and then reverse transcribed using the commercially available kit with random primers. cDNA was diluted appropriately and used for PCR, GAPDH was used as an internal control. DNA sequences corresponding to the HD and PEST domains were amplified using nested PCR according to previous report [7], and then sequencing was performed by Biotechnology Company.

Real-time PCR was performed as triplicate using SYBR Premix EX Taq (TAKARA) with an ABI PRISM 7300 real-time PCR system (Applied Biosystems, Life Technologies, Carlsbad, CA) with β-actin as the reference control. Primers used for quantitative RT-PCR are listed in Additional file 5: Table S2.

### Cell culture and transfection

Jurkat cells (ATCC, Rockville, MD) were grown in RPMI 1640 supplemented with 10% fetal calf serum, 2 mM L-glutamate, 100 U/ml penicillin, and 100 μg/ml streptomycin at 37°C in saturated humidity with 5% CO_2_. HeLa and Cos7 cells (ATCC) were maintained in Dulbecco’s modified Eagle medium (DMEM) containing the supplements mentioned above.

HeLa and Cos7 cells were transfected using Lipofectamine 2000 (Invitrogen) according to the recommended protocol. Jurkat cells (1 × 10^6^) were transfected with a Nucleofector Kit V (Amaxa-Lonza, Cologne, Germany) using a Nucleofector I (program X-01) following the manufacturer’s optimized protocol.

### Reporter assays

HeLa or Cos7 cells were cultured in 24-well plates and transfected with 5 ng phRL-TK (Promega, Madison, WI), 80 ng pGa981-6 reporter plasmid [17], 200 ng pEF-BOS-Myc-NIC, and serial amounts (100, 300, and 500 ng) of plasmids carrying FHL1C or various truncates of FHL1C. The cells were harvested at 48 h post-transfection, and cell extracts were assayed for luciferase activity using a Gloma X™ 20/20 Luminometer (Promega). The luciferase activity was normalized to Renilla luciferase activity.

### Flow cytometric analyses of cell cycle progression and apoptosis

Jurkat cells were resuspended in PBS and fixed in 70% ethanol on ice for 2 h. The cells were then stained with 20 mg/ml propidium iodide (PI) in PBS containing 0.1% Triton X-100 and 0.2 mg/ml RNase A for 30 min on ice. The cells were analyzed by a FACSCalibur flow cytometer (BD Immunocytometry Systems, San Jose, CA). Data were analyzed with CellQuest software.

Cell viability was routinely detected by trypan blue exclusion. Apoptosis was determined by staining with Annexin V-APC (eBiosciences, San Diego, CA) according to the manufacturer’s protocol, followed by flow cytometric analysis.

### Co-immunoprecipitation and western blotting

pEGFP-FHL1C and pCMV-Myc-RBP-J were transfected into HeLa cells. Co-immunoprecipitation was performed as described previously [17] with an anti-Myc antibody (9E10; Santa Cruz Biotechnology, Santa Cruz, CA). Western blotting was performed with anti-FHL1 (ProteinTech, Wuhan, China) or anti-Myc antibodies.

Western blotting analysis was performed routinely with primary antibodies including anti-AKT, anti-phospho-AKT (Signalway Antibody, Pearland, TX), anti-p50 (3354R-100; BioVision, Mountain View, CA), or anti-β-actin (Sigma-Aldrich, St. Louis, MO, USA). Anti-rabbit IgG and anti-mouse IgG (Boster BioTec, Shanghai, China) were used as secondary antibodies. Anti-c-Rel, anti-IκBα antibodies were purchased from Eptiomics (Abcam, Burlingame, CA). An anti-caspase 3 antibody (H-277), anti-GFP antibody, normal goat IgG, and normal rabbit IgG were purchased from Santa Cruz Biotechnology.

### Fractionation of subcellular components

Jurkat cells were washed twice with PBS at 4°C and then resuspended and incubated in buffer A (10 mM Hepes, 1.5 mM MgCl_2_, 10 mM KCl, 0.5 mM DTT, and a protease inhibitor cocktail) for 30 min on ice. After centrifugation at 4000 rpm for 20 min at 4°C, cytosolic fractions were collected, and the pellets were washed once in buffer A, resuspended in 1% NP-40 lysis buffer (10 mM Tris–HCl, pH 7.8, 0.5 mM EDTA, 250 mM NaCl, and the protease inhibitor cocktail), and then incubated for an additional 30 min on ice. After centrifugation at 10000 rpm for 15 min at 4°C, the nuclear factions were collected. Equal amounts of each fraction were analyzed by SDS-PAGE, followed by western blotting with the appropriate antibodies.

### Hoechst staining

Cells were washed twice with PBS, fixed in 70% ethanol for 20 min, and then washed again with PBS. Hoechst diluted at 1:10,000 (final concentration: 0.12 μg/ml) was added to cells followed by incubation in the dark for 15 min. The cells were washed with PBS and visualized under a fluorescence microscope (BX51; Olympus, Tokyo, Japan).

### Transmission electron microscopy (TEM)

Sample preparation and observation under a transmission electron microscope were performed as described previously [18].

### Statistical analysis

Data were analyzed with SPSS version 12.0 software. Results were expressed as the mean ± SD. Comparisons between groups were performed with the unpaired Student’s t-test. A P-value of less than 0.05 was considered statistically significant.

## Results

### FHL1C is down-regulated in PBMCs from T-ALL patients

**Figure 1:**
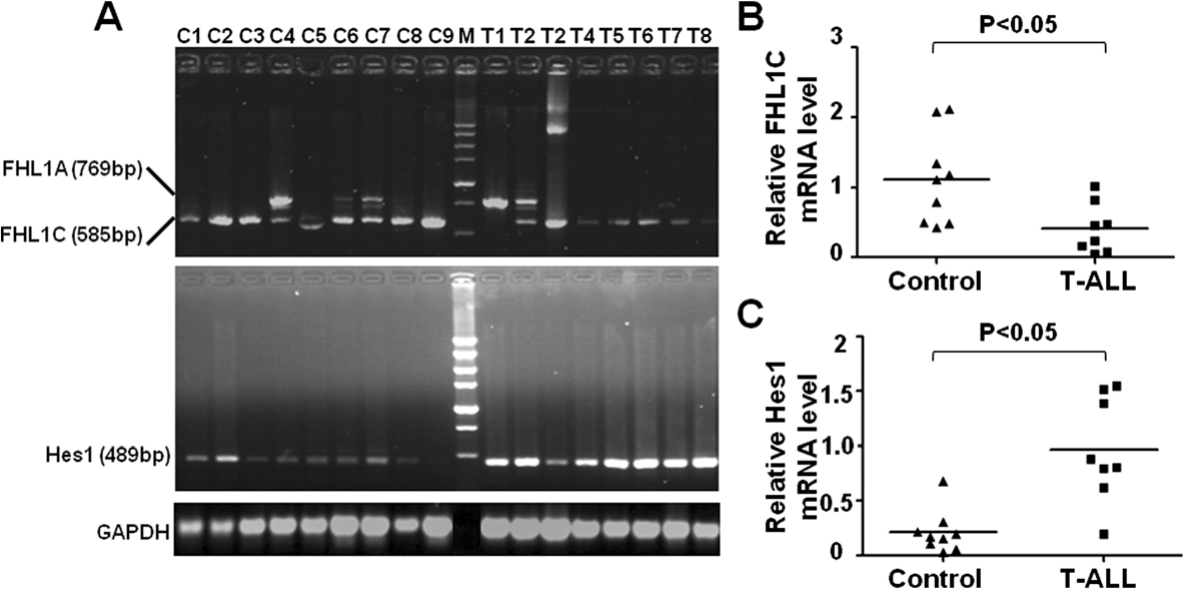
**Expression of FHL1C and Hes1 was detected in T-ALL patients and healthy donors. (A)** RT-PCR analysis of FHL1C and Hes1 mRNA in PBMCs from 8 T-ALL patients and 9 healthy donors, with GAPDH as an internal control. **(B, C)** Relative mRNA levels of FHL1C **(B)** and Hes1 **(C)** to GAPDH in PBMCs from T-ALL patients and controls were compared. The horizontal lines indicated median expression levels.

FHL1C/KyoT2 has been shown to be a negative regulator of the Notch pathway by competing with NIC for binding to RBP-J in vitro. To assess the relevance of FHL1C in T-ALL, we examined FHL1C mRNA expression in PBMCs from eight T-ALL patients and nine healthy donors as controls by RT-PCR. We found that FHL1C mRNA expression was significantly lower in PBMCs from T-ALL patients compared with that in PBMCs from healthy individuals (P < 0.05) (Figure 1A, upper panel and B). Because Hes1 is the main downstream target gene of activated Notch signaling in T-ALL [19], we also detected Hes1 mRNA expression in T-ALL and healthy individuals. The result showed that Hes1 mRNA expression was significantly higher in T-ALL samples than that in healthy individuals samples (Figure 1A, lower panel and C). These results indicate that FHL1C expression is down-regulated in the PBMCs of T-ALL patients.

### Overexpression of FHL1C induces apoptosis of T-ALL cells

**Figure 2:**
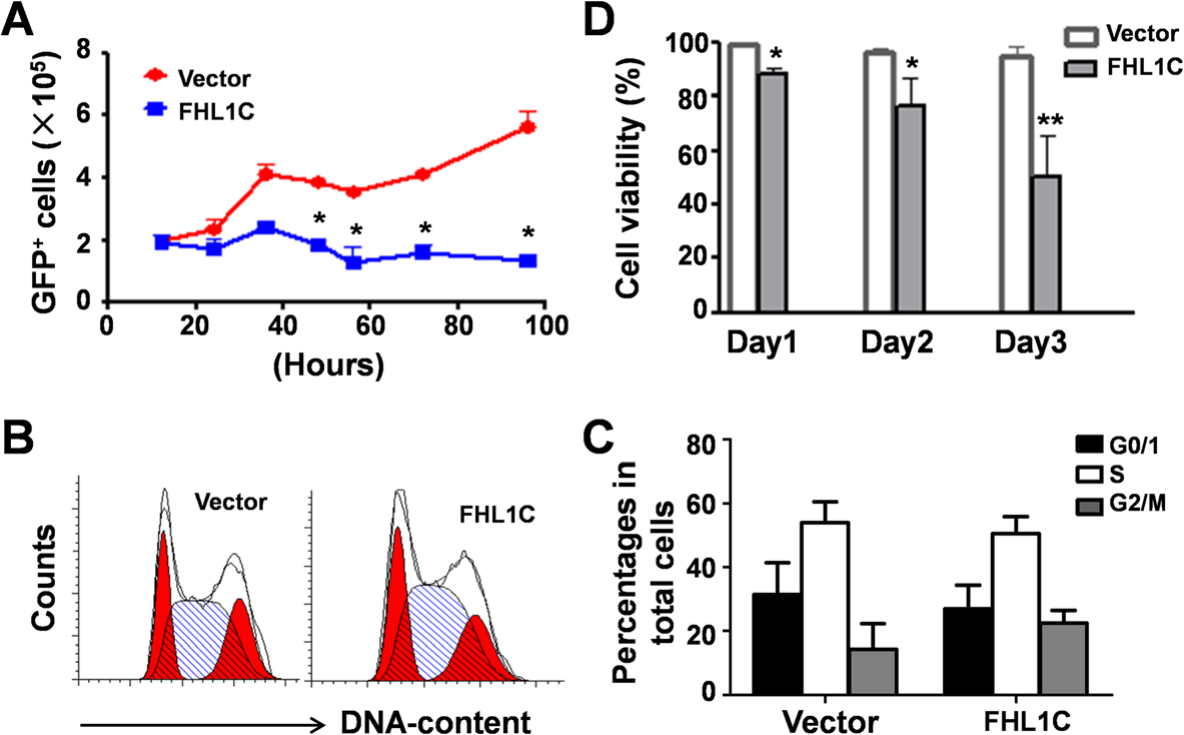
**Overexpression of FHL1C in Jurkat cells resulted in reduced cell viability. (A)** Jurkat cells (5 × 10^6^) were transfected with pEGFP or pEGFP-FHL1C by using the Nucleofection method. The numbers of viable EGFP-positive cells were determined every 12 h by cell counting and FACS analysis. **(B, C)** Cell cycle progression of Jurkat cells in **(A)** was determined 48 h post-transfection by FACS after PI staining **(B)**. Cells in each phase were compared between the two groups **(C)**. **(D)** Total viability of Jurkat cells transfected in **(A)** was monitored by using trypan blue exclusion assay. Bars = means ± S.D (n = 3), *P < 0.05, **P < 0.01.

To examine the role of FHL1C in T-ALL, we transiently overexpressed FHL1C in Jurkat cells, a human T-ALL cell line bearing Notch1 activation mutations. FHL1C was fused to EGFP at the N-terminus and introduced into Jurkat cells by electroporation. As determined by flow cytometric and western blotting analyses, EGFP expression showed that highly efficient transfection was achieved in both empty vector and pEGFP-FHL1C-transfected Jurkat cells (Additional file 6: Figure S1A and S1B). We monitored cell growth after transfection and found that the number of EGFP^+^ Jurkat cells transfected with pEGFP increased steadily, whereas the number of EGFP^+^ Jurkat cells transfected with pEGFP-FHL1C did not increase significantly and decreased gradually at 36 h post-transfection (Figure 2A). This observation suggested that overexpression of FHL1C caused cell growth arrest and/or cell death in Jurkat cells.We first examined the cell cycle progression of Jurkat cells transfected with pEGFP or pEGFP-FHL1C. The results showed no remarkable difference in the cell cycle distribution between the two groups, although the number of cells overexpressing FHL1C exhibited a slight increase in G2/M phase (Figure 2B and C). We next determined cell viability after transfection. We found that the percentage of viable cells decreased continuously among Jurkat cells after transfection with pEGFP-FHL1C, suggesting that overexpression of FHL1C might result in cell death (Figure 2D).

**Figure 3:**
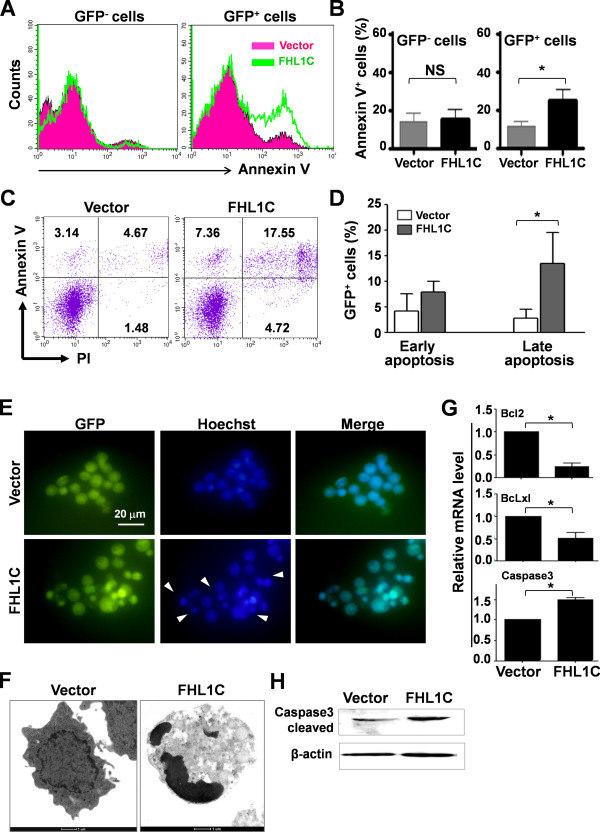
**FHL1C overexpression induced apoptosis in Jurkat cells. (A)** Jurkat cells were transiently transfected with pEGFP or pEGFP-FHL1C by using the Nucleofection method. Apoptosis in the GFP^−^ and GFP^+^ fractions of cells was determined by AnnexinV staining followed by FACS 48 h post-transfection. **(B)** Percentages of apoptotic cells (Annexin V^+^) in GFP^−^ and GFP^+^ cell fractions in **(A)** were compared. **(C)** Jurkat cells were transfected with pEGFP or pEGFP-FHL1C by using the Nucleofection method. Early and late apoptotic cells were depicted 48 h post-transfection by using Annexin V and PI staining followed by FACS. **(D)** GFP^+^ cells in early and late apoptotic phases in **(C)** were compared. **(E)** Jurkat cells were transiently transfected with pEGFP or pEGFP-FHL1C by using the Nucleofection method. Cells were stained with Hoechst 24 h post-transfection and nuclei were observed under a fluorescence microscope. Arrow heads indicate Hoechst-positive apoptotic nuclei. **(F)** Typical cell apoptosis in **(E)** was depicted under TEM. Intact cell membrane, organelles and normal nuclear morphology were observed in vector-transfected cells, whereas incomplete membrane and condensed nuclei were observed in cells overexpressing FHL1C (magnification, × 9900). **(G)** Total RNA was prepared from cells in **(E)** 24 h post-transfection. The mRNA levels of the apoptosis-related molecules were determined by real time RT-PCR, with β-actin as a reference. **(H)** Cell lysates were prepared from cells in **(E)** 24 h post-transfection. The level of Caspase3 was determined by Western blot analysis. Bars = means ± S.D (n = 3), *P < 0.05; NS, not significant.

Next, we directly estimated apoptosis after overexpression of FHL1C. Jurkat cells were transfected as described above, and apoptosis was determined by flow cytometric analysis with annexin-V and PI staining. In the GFP^+^ (transfected) cell population, there was a significant increase of annexin-V^+^ cells among the pEGFP-FHL1C-transfected Jurkat cells compared with that among the pEGFP-transfected Jurkat cells, suggesting that overexpression of FHL1C induced apoptosis in Jurkat cells (Figure 3A and B). Annexin-V and PI staining distinguishes early apoptotic (annexin V^+^PI^−^) and late apoptotic (annexin V^+^PI^+^) cells. As Figure 3C and D were shown, overexpression of FHL1C resulted in an increase of both early and late apoptotic cells among Jurkat cells. We also examined the morphology of Jurkat cells transfected with pEGFP or pEGFP-FHL1C by Hoechst staining (Figure 3E) and TEM (Figure 3F). The results confirmed that there were more apoptotic cells with condensed nuclei among Jurkat cells overexpressing FHL1C (Figure 3E and F). At the molecular level, overexpression of FHL1C in Jurkat cells reduced the expression of anti-apoptosis molecules, including Bcl-2 and Bcl-x1, and increased expression of the apoptosis-related molecule caspase 3 (Figure 3G and H). These results strongly suggest that overexpression of FHL1C induces apoptosis of T-ALL cells.

### FHL1C induces apoptosis of Jurkat cells through suppression of RBP-J-mediated transactivation

Similar to its murine homolog KyoT2, FHL1C also possesses a C-terminal RBP^motif^, suggesting that FHL1C interacts with RBP-J and suppresses RBP-J-mediated transactivation. To confirm an interaction between FHL1C and RBP-J, we performed co-immunoprecipitation. HeLa cells were co-transfected with expression vectors for Myc-tagged RBP-J (pCMV-Myc-RBP-J) and EGFP-tagged FHL1C (pEGFP-FHL1C), and immunoprecipitation was performed with an anti-Myc antibody. Co-precipitated proteins were detected using an anti-FHL1 antibody by western blotting analysis. The results showed that GFP-FHL1C was well co-precipitated with RBP-J (Additional file 7: Figure S2A), suggesting that FHL1C interacts with RBP-J. Furthermore, we performed reporter assays using HeLa and Cos7 cells by transfection with pEGFP-FHL1C and a NIC expression vector. As a result, overexpression of FHL1C suppressed transactivation of the reporter harboring RBP-J-binding sites by NIC in a dose-dependent manner (Additional file 7: Figure S2B). This result demonstrated that FHL1C suppresses RBP-J-mediated transactivation by competing with NIC.

**Figure 4:**
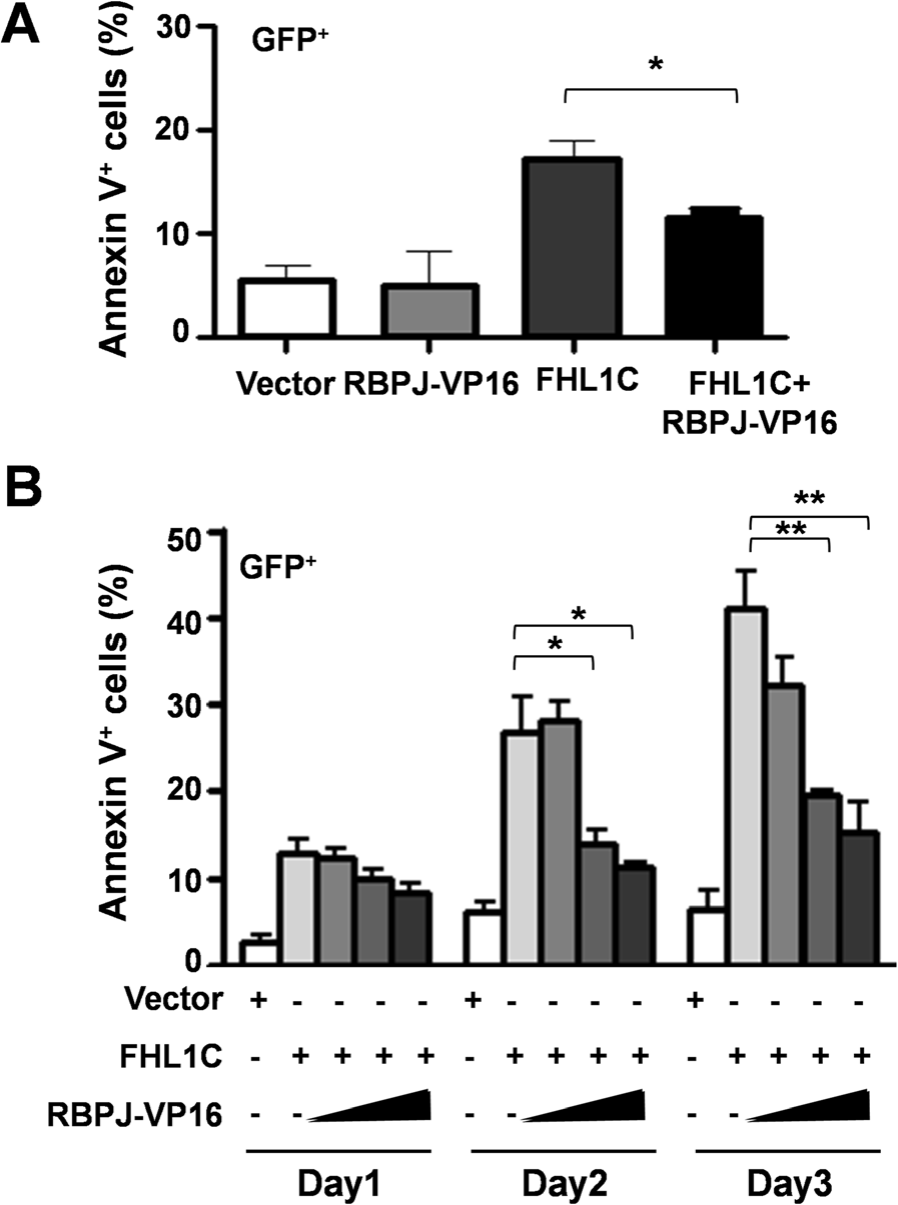
**FHL1C induced apoptosis of Jurkat cells through repressing RBP-J. (A)** Jurkat cells were transiently transfected with pEGFP, pCMX-VP16-RBP-J, pEGFP-FHL1C or pEGFP-FHL1C plus pCMX-VP16-RBP-J. The percentage of apoptotic (Annexin V^+^) cells in EGFP^+^ cell population was measured 24 h after transfection. **(B)** Constitutively active RBP-J blocked FHL1C-induced apoptosis in Jurkat cells. Jurkat cells were transiently transfected with 1 μg of pEGFP-FHL1C alone or in combination with increasing amounts (0.2, 0.5, 1.0 μg) of pCMX-VP16-RBP-J. Cell apoptosis was measured by Annexin V staining on different days after transfection. The percentages of apoptotic (Annexin V^+^) cells in EGFP^+^ cell population were shown. Bars = means ± S.D (n = 3), *P < 0.05, **P < 0.01.

We next determined whether FHL1C induced apoptosis of Jurkat cells through suppression of RBP-J-mediated transactivation by overexpressing RBP-J-VP16, a constitutively activated RBP-J [20]. Jurkat cells were transfected with pEGFP-FHL1C alone or co-transfected with pEGFP-FHL1C and pCMX-VP16-RBP-J, followed by analysis of apoptosis. The results showed that Jurkat cells did not undergo apoptosis after transfection with pCMX-VP16-RBP-J alone, and overexpression of FHL1C alone induced apoptosis, which was consistent with the results shown above. Co-transfection of cells with vectors carrying FHL1C and RBP-J-VP16 resulted in efficient attenuation of the FHL1C-induced apoptosis (Figure 4A). This effect was proportional to the amount of RBP-J-VP16 (Figure 4B). These data suggest that constitutively activated RBP-J protects Jurkat cells from FHL1C-induced apoptosis, most likely through constitutive activation of Notch target genes.

### The C-terminal RBP^motif^ of FHL1C is sufficient to induce apoptosis of Jurkat cells

FHL1C/KyoT2 is composed of two N-terminal LIM domains and a 27 amino acid RBP^motif^ at the C-terminus [21]. To determine which domain of FHL1C is critical for FHL1C-induced apoptosis of Jurkat cells, various EGFP fusion proteins in which EGFP was fused to full-length FHL1C, LIM1R, LIM2R, or RBP^motif^ were transfected into HeLa cells and then visualized under a confocal fluorescence microscope. As a result, these fusion proteins showed similar subcellular localization (Additional file 2: Figure S3A and S3B). Next, we examined the effect of these fusion proteins on RBP-J-mediated transactivation using a reporter assay. The results showed that all of the fusion proteins exhibited a transcription suppression effect on RBP-J-mediated transactivation of the reporter gene (Additional file 2: Figure S3C), although the full-length FHL1C fusion protein had the strongest activity.

**Figure 5:**
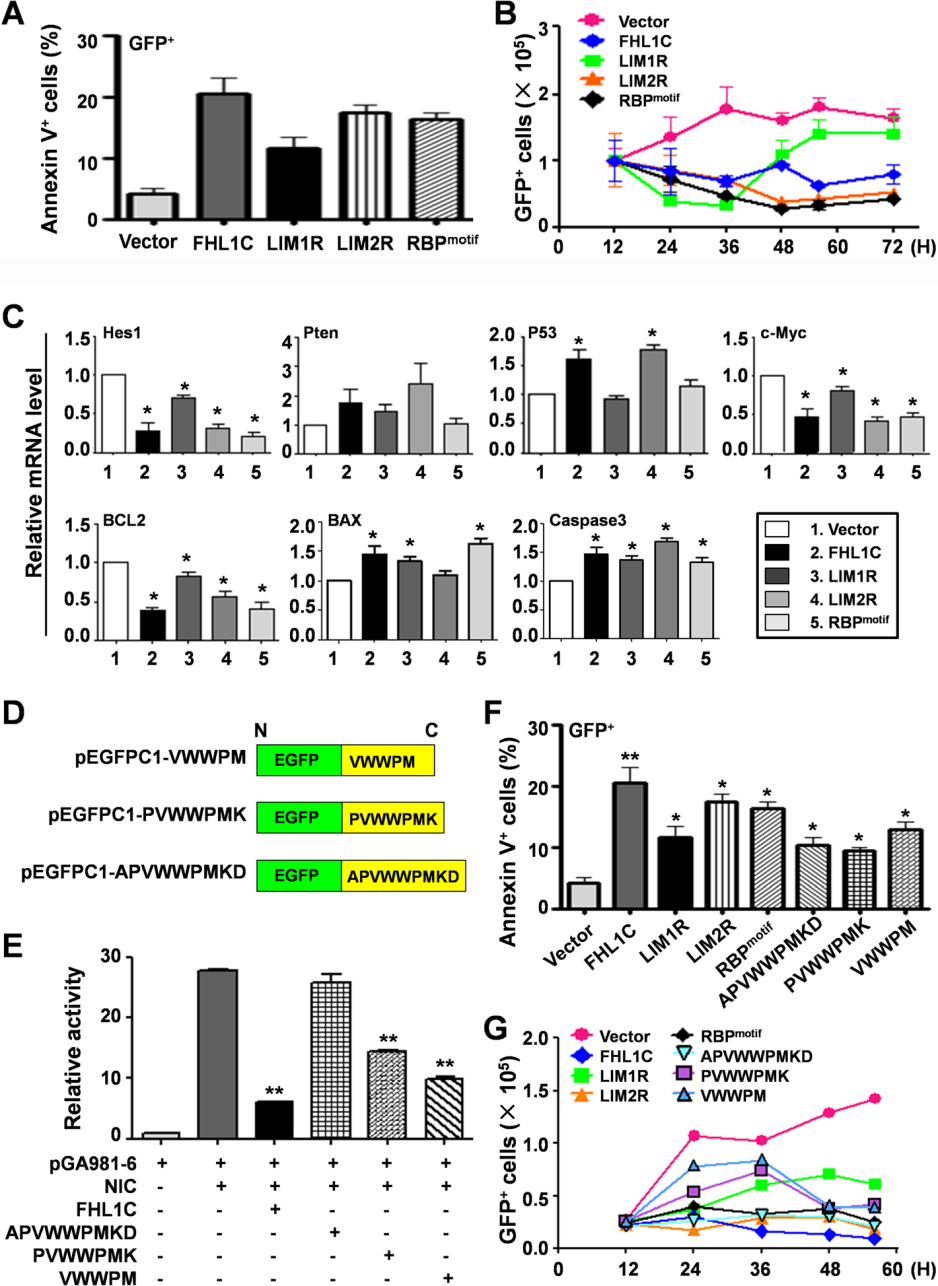
**The RBP-J-binding motif was sufficient to induce apoptosis in Jurkat cells. (A)** Full length and differentially truncated FHL1C (Additional file 7: Figure S3A) were inserted into pEGFPC1 in frame, and were used to transfect Jurkat cells. The cells were analyzed by Annexin V staining followed by FACS 48 h post-transfection. The percentages of apoptotic (Annexin V^+^) cells in the EGFP^+^ cell population were determined. **(B)** Jurkat cells were transiently transfected with plasmids as in **(A)**. The numbers of EGFP^+^ cells were counted at different time points after transfection. **(C)** Jurkat cells were transiently transfected with plasmids as in **(A)**. Cells were harvested 48 h post-transfection for RNA extraction. The mRNA expression levels of Hes1, Pten, Myc, p53, Bcl2, Bax, and Caspase3 were detected by qRT-PCR, with β-actin as a reference. **(D)** The core sequences with different length of the RBP-J-binding motif in FHL1C were fused to the 3′ terminus of EGFP in frame, to construct plasmids expressing EGFP with RBP-J-binding motif at the C-terminus. **(E)** EGFP containing RBP-J-binding motif inhibited NIC-mediated transactivation of RBP-J specific reporter construct. HeLa cells were transfected with different plasmids as indicated, and luciferase activity in the cell lysates was examined 48 h after transfection. **(F)** Jurkat cells were transiently transfected with plasmids as indicated. The cells were analyzed by Annexin V staining followed by FACS 48 h after the transfection. The percentages of apoptotic (Annexin V^+^) cells in the EGFP^+^ cell population were determined. **(G)** Jurkat cells were transiently transfected with plasmids as indicated. The numbers of GFP^+^ cells were counted at different time points after transfection. Bars = means ± S.D (n = 3), *P < 0.05, **P < 0.01.

We next evaluated the ability of these fusion proteins to induce apoptosis of Jurkat cells. Jurkat cells were transfected with each of the constructs, and apoptosis was assessed at 24 h post-transfection. We found that transfection of each construct induced apoptosis of Jurkat cells (Figure 5A). The number of GFP^+^ cells decreased continuously after transfection, except for EGFP-LIM1R-overexpressing cells that showed a decrease in cell number before 36 h post-transfection followed by an increase in the number of GFP^+^ cells (Figure 5B). We next examined the mRNA expression of critical downstream genes of Notch signaling, which are involved in T-ALL cells including Hes1 [19], Pten [22,23], p53 [24], and c-Myc [25,26], and apoptosis-related genes Bcl2, BAX [27], and caspase 3 [28]. The results showed that all of the fusion proteins down-regulated the expression of Hes1 and c-Myc, but EGFP-LIM1R only showed a mild effect. Consistent with the FHL1C-induced apoptosis, overexpression of these fusion proteins up-regulated apoptosis-promoting molecules while down-regulated apoptosis-inhibiting molecules (Figure 5C). These results suggest that the RBP^motif^ of FHL1C is sufficient to induce apoptosis of Jurkat cells.

These results raised the possibility of developing small peptides to disrupt Notch signaling in T-ALL cells. Therefore, as the first step, we determined which sequence in the RBP^motif^ of FHL1C could induce Jurkat cell apoptosis. Oligonucleotides encoding various lengths of the RBP^motif^ were synthesized, fused to the C-terminus of EGFP (Figure 5D), and then overexpressed in Jurkat cells by transfection. All constructs exhibited suppression of Notch-mediated transcriptional activation in reporter assays, but the construct carrying EGFP fused to the VWWPM motif showed suppression comparable with that of full-length FHL1C (Figure 5E). We next examined apoptosis by annexin-V staining. In the GFP^+^ cell population, overexpression of EGFP-VWWPM efficiently induced apoptosis of Jurkat cells, although the other two fusion proteins had similar effects (Figure 5F). Consistently, overexpression of EGFP fused to various lengths of the RBP^motif^ resulted in a reduction of the number of transfected GFP^+^ Jurkat cells (Figure 5G). These results suggest that a minimal RBP-J-binding sequence composed of five amino acids (VWWPM) is enough to induce apoptosis of T-ALL cells.

### Overexpression of FHLIC inhibits downstream genes and key pathways of notch signaling in T-ALL progression

**Figure 6:**
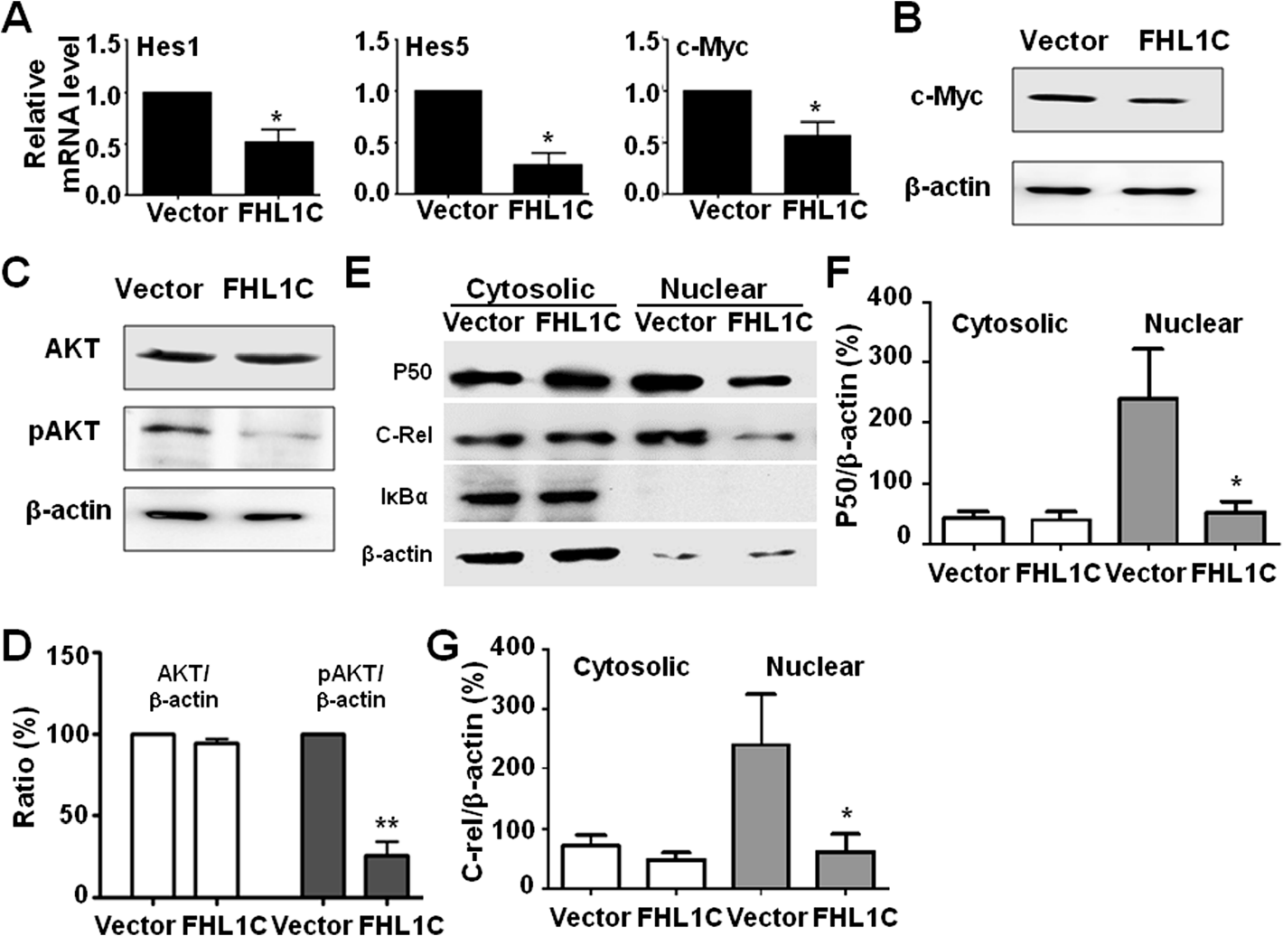
**Overexpression of FHL1C induced apoptosis of Jurkat cells involving multiple effectors and pathways. (A)** Jurkat cells were transfected with pEGFP or pEGFP-FHL1C by using the Nucleofection method. The cells were harvested 48 h post-transfection, and the mRNA levels of Hes1, Hes5 and c-Myc were detected by real time RT-PCR, with β-actin as a reference. **(B)** Jurkat cells were transfected as in **(A)**. The protein level of c-Myc was determined by using Western blotting. **(C,D)** Cell lysates were prepared from Jurkat cells transfected with pEGFP or pEGFP-FHL1C for 48 h. AKT and phosphorylated AKT (pAKT) were analyzed by Western blotting **(C)**. The relative levels of AKT and pAKT were quantified and compared, with β-actin as an internal control **(D)**. **(E-G)** Jurkat cells were transfected with pEGFP or pEGFP-FHL1C by using the Nucleofection method. Cells were harvested 24 h post-transfection, and the cytosolic and nuclear extracts were fractioned. P50, c-Rel and IκB were determined by Western blotting **(E)**. The relative levels of P50 **(F)** and c-Rel **(G)** were quantified and compared, with β-actin as an internal control. Bars = means ± S.D (n = 3), *P < 0.05, **P < 0.01.

To explore whether FHL1C-mediated apoptosis of Jurkat cells is associated with attenuation of Notch signaling, we first examined expression of the critical downstream genes of the Notch pathway involved in T-ALL progression using quantitative RT-PCR and western blotting. As a result, the mRNA levels of Hes1, Hes5, and c-Myc were significantly down-regulated by FHL1C overexpression (Figure 6A). The protein level of c-Myc was also reduced remarkably (Figure 6B). These data indicate that FHL1C regulates T-ALL progression by direct suppression of Notch1 target gene expression.

Furthermore, we examined the effects of FHL1C overexpression on the activation of PI3K/AKT and NF-κB by western blotting, which are critical pathways activated by Notch1 in T-ALL [29,30]. We found that overexpression of FHL1C in Jurkat cells reduced the phosphorylation of AKT (Figure 6C and D). Activation of NF-κB is closely associated with Notch1-dependent T-ALL. Therefore, we examined the levels of p50, c-Rel, and IκB in the cytosolic and nuclear fractions of FHL1C-overexpressing Jurkat cells by western blotting. The results showed that the levels of p50 and c-Rel decreased significantly in the nuclear fraction. IκB was found primarily in the cytosolic fraction and was also decreased slightly upon FHL1C overexpression (Figure 6E–G). This data suggest that FHL1C might down-regulate NF-κB activity by inhibiting nuclear translocation of p50 and c-Rel.

## Discussion

The identification of activating point mutations in Notch1 in more than 50% of T-ALL cases has spurred the development of therapies targeting the Notch1 signaling pathway for the treatment of T-ALL. To date, most of these efforts have focused on inhibiting the activity of γ-secretase, an enzyme that is essential for Notch receptor activation. Small molecule GSIs that inhibit γ-secretase activity have been tested in clinical trials and shown down-regulation of Notch1 target genes in T-ALL cells [7,31]. However, GSIs are not selective for Notch1 signaling and block other Notch receptors and physiological pathways requiring γ-secretase. Indeed, patients have developed marked fatigue and dose-limiting gastrointestinal toxicity in clinical trials of GSIs, because of the inhibition of Notch1 and Notch2 in intestinal crypt progenitors and/or stem cells, resulting in premature differentiation into goblet cells [32]. However, Real et al. subsequently showed that the gut toxicity can be ameliorated by combinatorial therapy using GSIs and glucocorticoids [12]. To avoid the side effects of GSIs, antibodies have been developed to specifically block the Notch1 receptor [33]. However, it has been demonstrated that the hotspot region of Notch1 mutations in T-ALL is the PEST domain located in the C-terminus of Notch1, which leads to delayed NIC degradation and thus prolonged Notch signaling. Therefore, these mutations are less sensitive to anti-Notch antibodies [30,34]. In addition, some tumor cells harboring chromosomal translocations or other genetic aberrations might not be suitable for antibody-mediated therapy [35]. In addition to PEST domain mutations, another region of Notch1 mutations in T-ALL is the NRR region including the LNR and HD domains, in which mutations lead to ligand hypersensitivity and ligand-independent activation [7]. Although anti-NRR antibodies have been developed, sustained treatment with these antibodies will likely cause vascular neoplasms [36]. More recently, Roti et al. demonstrated that inhibition of SERCA (sacro/endoplasmic reticulum Ca^2+^-ATPase) calcium pumps preferentially affects the maturation and activity of mutant Notch1 receptors, leading to enhanced clearance of the mutant Notch protein. Even if SERCA can be specifically targeted, such inhibition does not effect on T-ALL cells with activated Myc mutations or lacking NRR region [37].

The transactivation complex NIC-RBP-J-MAML1 is critical for signaling from Notch receptors, and is thus becoming a promising therapeutic target for T-ALL at the transcription level. Recently, Moellering et al. showed that SAHM1 suppresses the transcriptional complexes of Notch signaling. Treatment of leukemic cells with SAHM1 inhibits cell proliferation in vitro and in a Notch1-driven T-ALL mouse model without prominent gut toxicity [16]. In the current study, we found that overexpression of FHL1C induced apoptosis of the Jurkat T-ALL cell line in vitro. FHL1C overexpression down-regulated c-Myc expression and attenuated the PI3K/AKT pathway and NF-κB signaling. These mechanisms may be involved in the enhanced apoptosis of Jurkat cells overexpressing FHL1C (Additional file 8: Figure S4), and suggest that FHL1C may be another therapeutic target for T-ALL at the transcriptional level. Moreover, it has been shown that Pten plays an important role in negative regulation of PI3K/AKT signaling in T-ALL. However, because Jurkat cells lack active Pten protein expression, it is possible that FHL1C can suppress AKT by other mechanisms such as disruption of the NICD-P56^Lck^-PI3K complex [30,38,39]. Further studies are needed to investigate whether FHL1C can inhibit AKT activation through Pten in native T-ALL cells.

FHL1 is a member of the FHL protein family that contains four-and-a-half LIM domains. FHL1 family members interact with many proteins through their LIM domains, including transcription factors, enzymes, and cytoskeleton proteins. These proteins play important roles in cell differentiation and cytoskeleton formation. Recent studies have shown that FHL1 also has important functions in tumorigenesis and cancer progression. FHL1 expression is suppressed in a variety of tumors including lung cancer, breast cancer, brain tumors, and gastric cancer [40,41]. In contrast, some reports show that FHL1 is expressed at a high level in a squamous cell carcinoma cell line [42]. FHL1 is aberrantly expressed in most T-ALL cell lines, particularly those exhibiting deregulated TLX1/HOX11 expression after specific chromosome translocation [43]. In our study using PBMCs from T-ALL patients, we detected FHL1A expression in two cases, but the significance and underlying mechanism are unclear. We also detected significant down-regulation of FHL1C expression in PBMCs of T-ALL patient, accompanied by up-regulation of Hes1, a Notch target gene involved in T-ALL progression. These results suggest that FHL1C may be involved in T-ALL progression and can be used as a therapeutic target of the disease. However, the mechanism regulating FHL1C expression in T-ALL cells remains unknown, and whether FHL1C is involved in other cancers is unclear. In addition, although FHL1B (KyoT3) is another isoform of FHL1, which encodes a 34 kDa polypeptide containing the same RBP^motif^ found in FHL1C [44,45], we did not detect FHL1B expression in T-ALL patients or normal healthy individuals.

FHL1C/KyoT2 encodes a 22 kDa protein sharing the two N-terminal LIM domains with FHL1A, and a 27 amino acid RBP-J-binding region at the C-terminus generated by alternative splicing. FHL1C/KyoT2 may participate in suppression of RBP-J-mediated Notch signaling by two mechanisms: competing with NIC for binding to RBP-J [17,46] or recruitment of co-repressors. The LIM domain is a protein interaction interface that is involved in linking proteins with the actin cytoskeleton and/or transcriptional machinery [47,48]. Our previous studies have shown that KyoT2 might suppress RBP-J-mediated Notch transactivation by recruiting the Polycomb suppression complex including RING1 and HPC2 through the LIM domains. Furthermore, KyoT2-mediated repression of Notch transactivation may be regulated by sumoylation involving PIAS1 [17,46,49]. In this study, we showed that overexpression of FHL1C induced apoptosis of Jurkat cells. Through a series of structure-function analyses, we found that such apoptosis was mainly mediated through the C-terminal RBP^motif^ of FHL1C, suggesting that competitive binding to RBP-J might be the major mechanism. Nevertheless, we cannot exclude the involvement of other interacting molecules. More importantly, we found that a minimal pentapeptide motif, VWWPM, suppressed RBP-J-mediated Notch activation and induced apoptosis of T-ALL cells at a relatively high efficiency. We expect that this peptide sequence will benefit future Notch-targeted therapies of T-ALL.

## Conclusions

Taken together, our study revealed that overexpression of FHL1C induces Jurkat cell apoptosis. This finding may provide new insights into the design of new Notch inhibitors based on FHL1C to treat T-ALL in the future.

## Abbreviations

FHL1C: Four-and-half LIM domain protein1C; GSI: γ-secretase inhibitor; Hes: Hairy and enhancer of split; NIC: Notch intracellular domain; MAML1: Mastermind-like1; DN-MAML1: Dominant-negative MAML1; PBMCs: Peripheral blood mononuclear cells; qPCR: Quantitative PCR; RAM: RBP-J association molecule; RBP-J: Recombination signal binding protein-J; SAHM1: Stapled α-helical peptides derived from MAML1; SERCA: Sacro/endoplasmic reticulum Ca^2+^-ATPase.

## Competing interests

The authors declare that they have no competing interests.

## Authors’ contributions

FW, WK, ZJL, YHC, LSZ, LY, LL and HSY performed the research, QHY and HH designed the research study, FW, WK, ZJL, QHY and HH analyzed data, FW, QHY and HH wrote the paper, LYM provided advice and critical discussion on the project. All authors read and approved the final manuscript.

## Pre-publication history

The pre-publication history for this paper can be accessed here:

http://www.biomedcentral.com/1471-2407/14/463/prepub

## Supplementary Material

Additional file 1: Table S1 The sequences of PCR primers used in the study. Click here for file

Additional file 2: Figure S3 Construction of different truncates of FHL1C containing the RBP-J-binding motif. **(A)** Schematic diagrams of constructs expressing EGFP fused with different truncated FHL1C. **(B)** Locations of these different truncates of FHL1C in HeLa cells. HeLa cells were transiently transfected with the indicated plasmids. The cells were stained with Hoechst 24 h post-transfection, and examined under a confocal microscope. Scale bars = 20 μm. **(C)** Full length and different truncated FHL1C were inserted into pEGFPC1 in frame, and were used to transfect HeLa cells with NIC-expressing vector and pGa981-6 (the reporter plasmid). Cells were harvested 48 h post-transfection, and luciferase activity in cell lysates was analyzed. Click here for file

Additional file 3: Table S3 Clinical characteristics of patients suffering from T-ALL. Click here for file

Additional file 4: Table S4 Clinical characteristics of healthy donors. Click here for file

Additional file 5: Table S2 The sequences of real time PCR primers. Click here for file

Additional file 6: Figure S1 Overexpression of EGFP-FHL1C fusion protein in Jurkat cells. **(A)** Jurkat cells (5 × 10^6^) were transfected with pEGFP or pEGFP-FHL1C by using the Nucleofection method. Cells were observed under a fluorescence microscope (upper) and analyzed by FACS (lower) 48 h post-transfection, the expression of EGFP or EGFP-FHL1C was determined by FACS respectively. **(B)** Cell lysates were prepared from Jurkat cells in **(A)**, and the expression of EGFP or EGFP-FHL1C was determined by Western blotting using anti-EGFP antibody, with β-actin as an internal control. Click here for file

Additional file 7: Figure S2 FHL1C interacted with RBP-J and inhibited Notch signaling. **(A)** HeLa cells were transfected with pEGFP-FHL1C and pCMV-Myc-RBP-J as indicated. Cell lysates were prepared 48 h post-transfection, and the interaction between FHL1C and RBP-J was determined by using co-immunoprecipitation. **(B)** HeLa and Cos7 cells were transfected with plasmids as indicated, and luciferase activities in cell lysates were examined 48 h post-transfection. Click here for file

Additional file 8: Figure S4 Potential molecular mechanism of FHL1C-mediated regulation of T-ALL progression. FHL1C represses Notch1-dependent T-ALL progression by suppressing critical downstream molecules and pathways of Notch signaling through RBP-J. Solid lines show tested signaling pathway. Dotted lines show untested signaling pathway. Click here for file
