# Supplementary material for: FHL1C induces apoptosis in notch1-dependent T-ALL cells through an interaction with RBP-J
Source: BMC Cancer. 2014 Jun 22;14:463. doi: 10.1186/1471-2407-14-463 (PMC4077834; doi:10.1186/1471-2407-14-463)
Supplement: Additional file 1: Table S1 — The sequences of PCR primers used in the study. [file 1471-2407-14-463-S1.doc]

**Table S1.** The sequences of PCR primers used in the study.

| Gene | Primer sequences |
| --- | --- |
| FHL1C-F | 5'-ATGGCGGAGAAGTTTGACTGCCACTACT-3' |
| FHL1C-R | 5'-TCACGGAGCATTTTTTGCAGTGGAAGCA-3' |
| LIM1-F | 5'-GCGAATTCTATGGCGGAGAAGTTTGACTG-3' |
| LIM1-R | 5'-GGGGGAGTCCTCCCGAGTGG-3' |
| RBPJ-F | 5'-GCGAATTCTATGGGTTTGGTAAAGGCTCC-3' |
| RBPJ-R | 5'-CCCGGGTCACGGAGCATTTTTTGCAG -3' |
| LIM2-F | 5'-GCGAATTCT ATGAAGTGCAAGGGGTGCTT-3' |
| hsa-HES1-F | 5'-AGTCTGAGCCAGCTGAAAACAC-3' |
| hsa-HES1-R | 5'-ACACCTTAGCCGCCTCTCCA-3' |
| hsa-GAPDH-F | 5'-ACCACAGTCCATGCCATCAC-3' |
| hsa-GAPDH-R  Notch1-HD-F1  Notch1-HD-R1  Notch1-HD-F2  Notch1-HD-R2  Notch1-PEST-F1  Notch1-PEST-R1  Notch1-PEST-F2  Notch1-PEST-R2 | 5'-TCCACCACCCTGTTGCTGTA-3'  5’-AGCCCCCTGTACGACCAGTA-3’  5’-CTTGCGCAGCTCCTCCTC-3’  5’-GACCAGTACTGCAAGGACCA-3’  5’-TCCTCGCGGCCGTAGTAG-3’  5’-GCAGCATGGCATGGTAGG-3’  5’-AACATGTGTTTTAAAAAGGCTCCTC-3’  5’-AAACATCCAGCAGCAGCAAA-3’  5’-CACAGGCGAGGAGTAGCTGTG-3’ |
