# Supplementary figures and images for: FHL1C induces apoptosis in notch1-dependent T-ALL cells through an interaction with RBP-J
Source: BMC Cancer. 2014 Jun 22;14:463. doi: 10.1186/1471-2407-14-463 (PMC4077834; doi:10.1186/1471-2407-14-463)

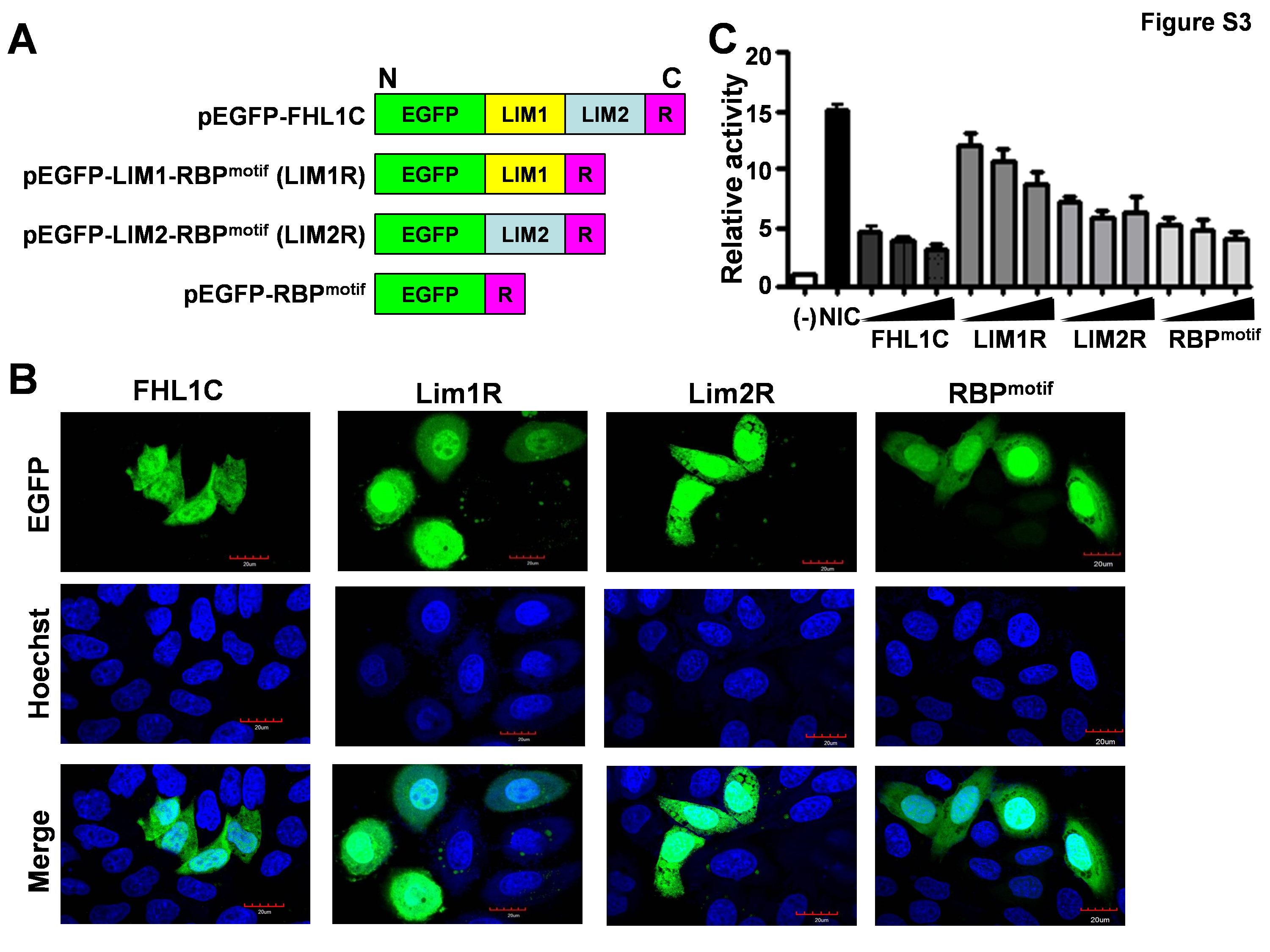

Supplement: Additional file 2: Figure S3 — Construction of different truncates of FHL1C containing the RBP-J-binding motif. (A) Schematic diagrams of constructs expressing EGFP fused with different truncated FHL1C. (B) Locations of these different truncates of FHL1C in HeLa cells. HeLa cells were transiently transfected with the indicated plasmids. The cells were stained with Hoechst 24 h post-transfection, and examined under a confocal microscope. Scale bars = 20 μm. (C) Full length and different truncated FHL1C were inserted into pEGFPC1 in frame, and were used to transfect HeLa cells with NIC-expressing vector and pGa981-6 (the reporter plasmid). Cells were harvested 48 h post-transfection, and luciferase activity in cell lysates was analyzed. [file 1471-2407-14-463-S2.tiff]

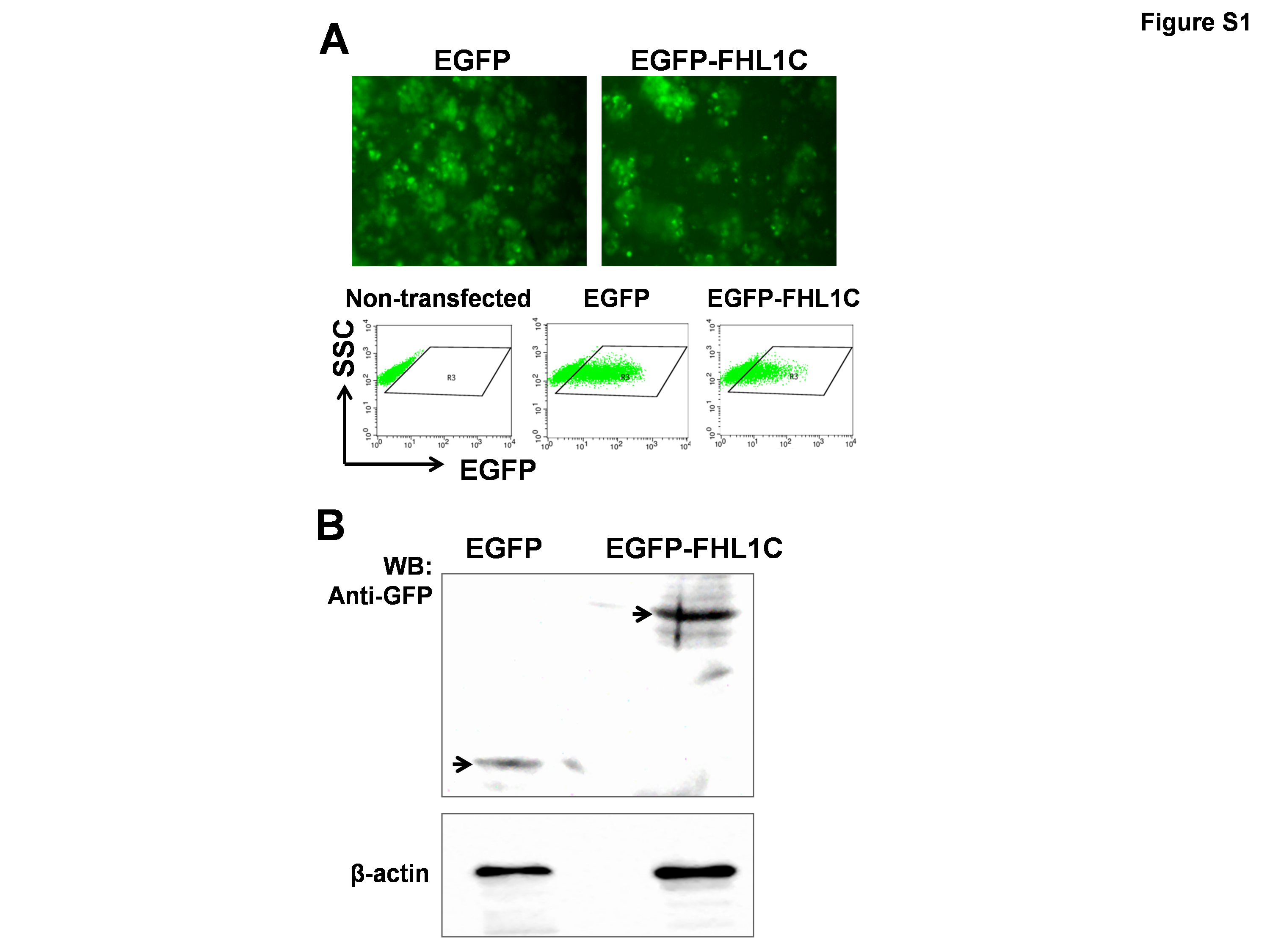

Supplement: Additional file 6: Figure S1 — Overexpression of EGFP-FHL1C fusion protein in Jurkat cells. (A) Jurkat cells (5 × 10^6^) were transfected with pEGFP or pEGFP-FHL1C by using the Nucleofection method. Cells were observed under a fluorescence microscope (upper) and analyzed by FACS (lower) 48 h post-transfection, the expression of EGFP or EGFP-FHL1C was determined by FACS respectively. (B) Cell lysates were prepared from Jurkat cells in (A), and the expression of EGFP or EGFP-FHL1C was determined by Western blotting using anti-EGFP antibody, with β-actin as an internal control. [file 1471-2407-14-463-S6.tiff]

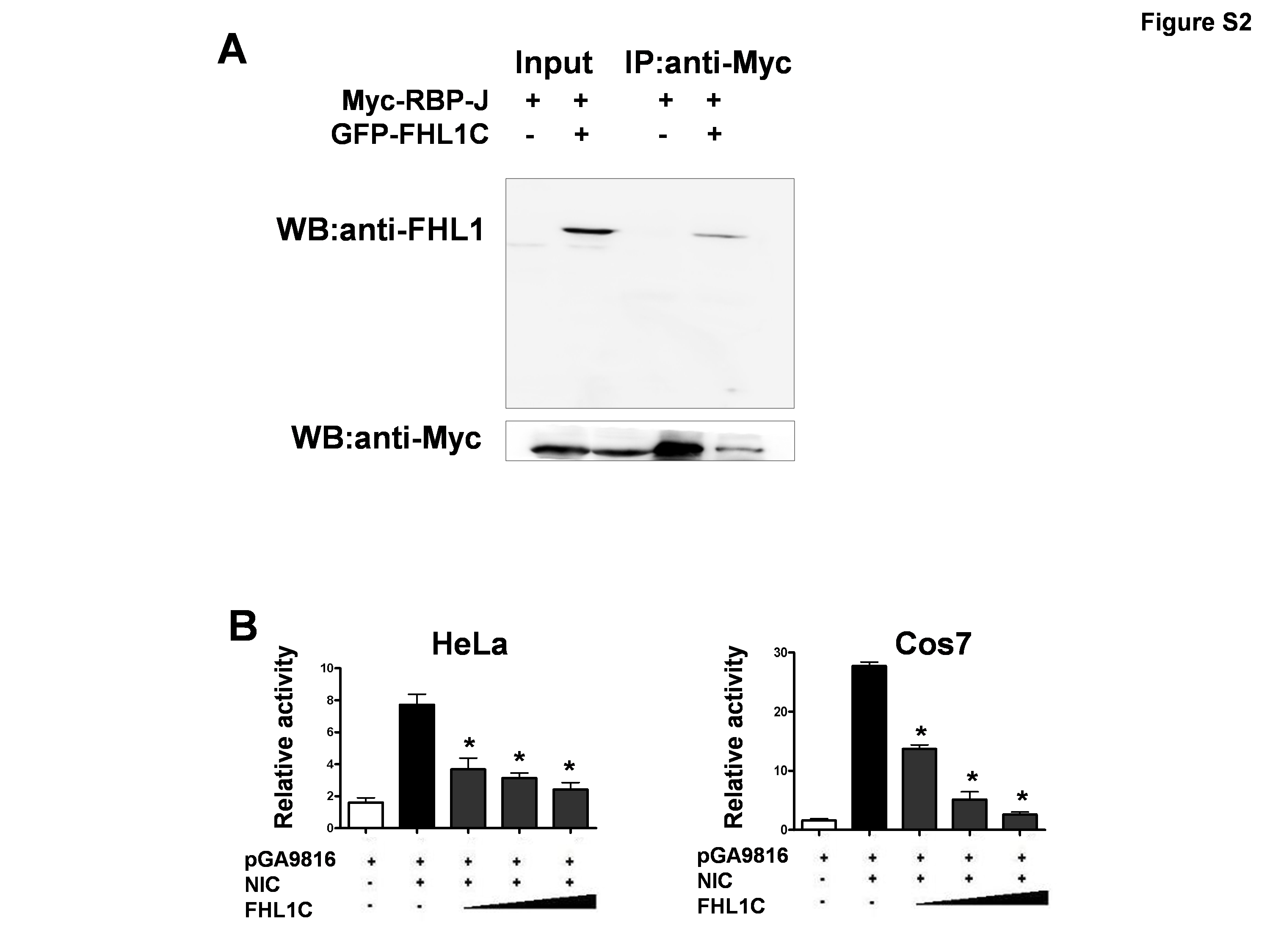

Supplement: Additional file 7: Figure S2 — FHL1C interacted with RBP-J and inhibited Notch signaling. (A) HeLa cells were transfected with pEGFP-FHL1C and pCMV-Myc-RBP-J as indicated. Cell lysates were prepared 48 h post-transfection, and the interaction between FHL1C and RBP-J was determined by using co-immunoprecipitation. (B) HeLa and Cos7 cells were transfected with plasmids as indicated, and luciferase activities in cell lysates were examined 48 h post-transfection. [file 1471-2407-14-463-S7.tiff]

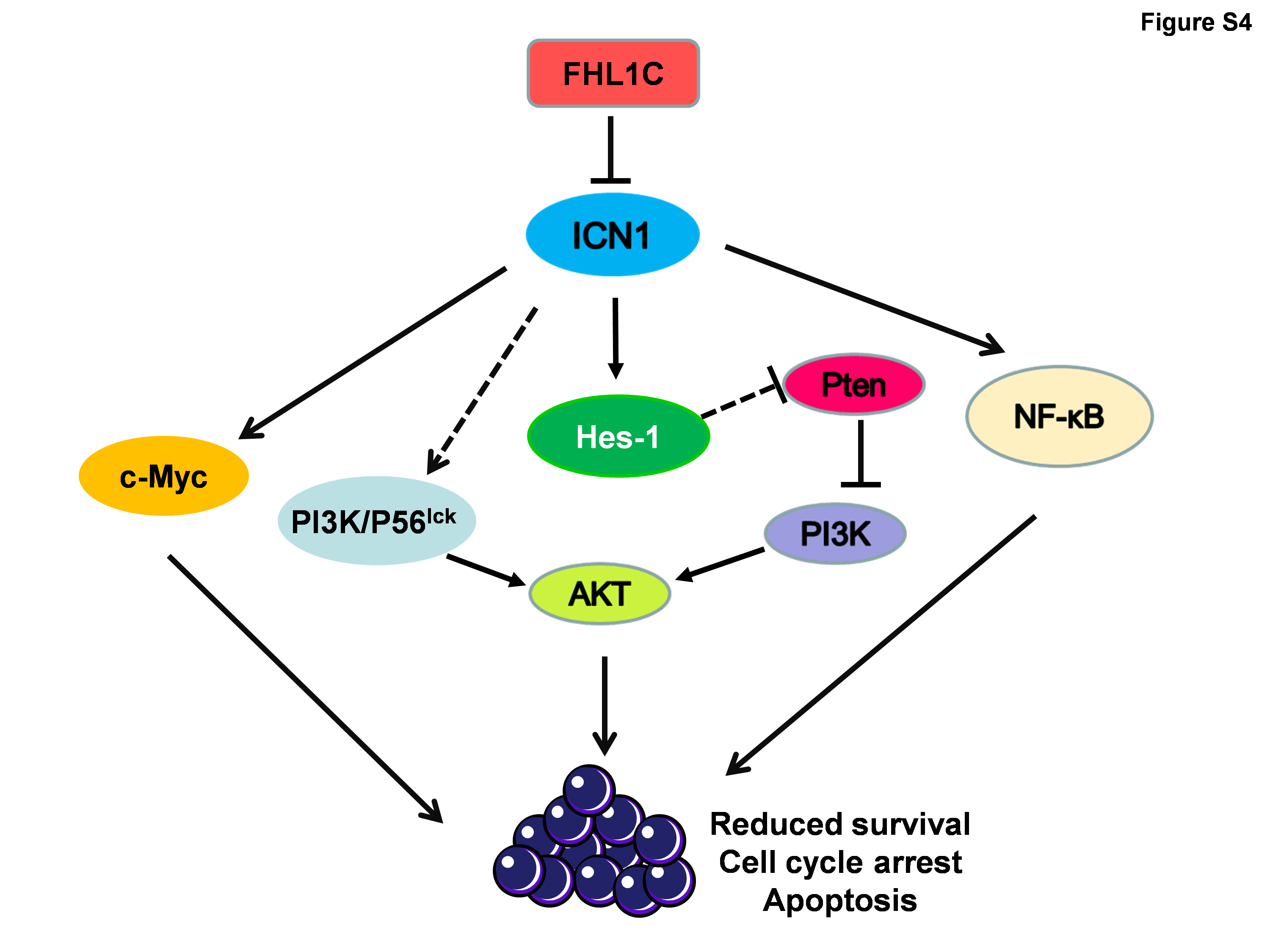

Supplement: Additional file 8: Figure S4 — Potential molecular mechanism of FHL1C-mediated regulation of T-ALL progression. FHL1C represses Notch1-dependent T-ALL progression by suppressing critical downstream molecules and pathways of Notch signaling through RBP-J. Solid lines show tested signaling pathway. Dotted lines show untested signaling pathway. [file 1471-2407-14-463-S8.tiff]
