# Supplementary material for: FHL1C induces apoptosis in notch1-dependent T-ALL cells through an interaction with RBP-J
Source: BMC Cancer. 2014 Jun 22;14:463. doi: 10.1186/1471-2407-14-463 (PMC4077834; doi:10.1186/1471-2407-14-463)
Supplement: Additional file 3: Table S3 — Clinical characteristics of patients suffering from T-ALL. [file 1471-2407-14-463-S3.doc]

**Table S3. Clinical characteristics of patients suffering from T-ALL**

| **Patient**  **No.** | **HB**  **(g/dl)** | **WBC count**  **(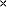 10^9^/l)** | **Platelet count**  **(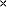 10^9^/l)** | **Bone marrow blast cells（%）** | **Immune**  **phenotype** | **Outcome of**  **treatment** | **EFS**  **（months）** | **OS**  **（months）** | **Notch**  **Mutations** |
| --- | --- | --- | --- | --- | --- | --- | --- | --- | --- |
| **1** | 6.8 | 20 | 120 | 90.15 | CD3 cCD3 CD5 CD7 CD10 CD38 CD79 HLA-DR | Death in CR | 8 | 17 | HD |
| **2** | 11.2 | 223 | 85 | 97.14 | CD2 CD4 cCD3 CD5 CD7 CD8 HLA-DR CD38 | Death in PR | 0 | 3 | HD |
| **3** | 7.2 | 125 | 35 | 85 | cCD3 CD5 CD7 CD10 CD11c CD78 | Alive in CR | 32 | 33 | HD |
| **4** | 12 | 52 | 110 | 81.3 | cCD3 CD7 CD13 CD33 CD34 CD38 CD79a HLA-DR | Alive in CR | 39 | 40 | HD |
| **5** | 6.2 | 234 | 90 | 38.92 | CD1a CD2 CD3 cCD3 CD5 CD7 CD13 CD38 | Death after relapse | 2 | 5 | HD |
| **6** | 5.0 | 300 | 60 | 83.5 | CD3 cCD3 CD5 CD4 CD7 CD8 CD10 CD23 CD38 CD79a | Death after non response | 0 | 3 | HD/PEST |
| **7** | 10.5 | 120 | 120 | 95.5 | cCD3 CD5 CD7 CD13 CD38 | Death after relapse | 2 | 7 | HD |
| **8** | 9.5 | 210 | 79 | 99 | CD3 cCD3 CD5 CD7 CD13 CD34 CD38 CD79a | Death after non response | 0 | 5 | HD |

CR, complete remission ;PR,partial remission ; EFS, Event-free survival ;OS,Overall survival;HD, heterodimerization domain;PEST, proline, glutamic acid, serine, threonine-rich domain.
