# Supplementary material for: FHL1C induces apoptosis in notch1-dependent T-ALL cells through an interaction with RBP-J
Source: BMC Cancer. 2014 Jun 22;14:463. doi: 10.1186/1471-2407-14-463 (PMC4077834; doi:10.1186/1471-2407-14-463)
Supplement: Additional file 4: Table S4 — Clinical characteristics of healthy donors. [file 1471-2407-14-463-S4.doc]

**Table S4.** Clinical characteristics of healthy donors

| **Parameter** | **Healthy donors(n=9)** |
| --- | --- |
| **Sex** |  |
| **Male** | 4 |
| **Female** | 5 |
| **Median age (range),years** | 23(5-42) |
| **HB(g/dl)** | 13.1(10-15.5) |
| **WBC count (range),х10^9^/l** | 6.3(4.5-8.2) |
| **Platelet count (range),х10^9^/l** | 210(145-285) |
