# Supplementary material for: FHL1C induces apoptosis in notch1-dependent T-ALL cells through an interaction with RBP-J
Source: BMC Cancer. 2014 Jun 22;14:463. doi: 10.1186/1471-2407-14-463 (PMC4077834; doi:10.1186/1471-2407-14-463)
Supplement: Additional file 5: Table S2 — The sequences of real time PCR primers. [file 1471-2407-14-463-S5.doc]

**Table S2. The sequences of real time PCR primers**

| Gene | Primer sequences |
| --- | --- |
| hsa-Hes1-F | 5'-GGACATTCTGGAAATGACAGTGA-3' |
| hsa-Hes1-R | 5'-AGCACACTTGGGTCTGTGCTC-3' |
| hsa-Hes5-F | 5'-CACCAGGACTACAGCGAAGGCTA-3' |
| hsa-Hes5-R | 5'-TGGAGCGTCAGGAACTGCAC-3' |
| hsa-CASP3-F | 5'-GACTCTGGAATATCCCTGGACAACA-3' |
| hsa-CASP3-R | 5'-CTGAGGTTTGCTGCATCGACA-3' |
| hsa-p53-F | 5'-AGAGCTGAATGAGGCCTTGGAA-3' |
| hsa- p53-R | 5'-GAGTCAGGCCCTTCTGTCTTGAAC-3' |
| hsa- Myc-F | 5'-GCAGCTGCTTAGACGCTGGA-3' |
| hsa-Myc-R | 5'-CGCAGTAGAAATACGGCTGCAC-3' |
| hsa- PTEN-F | 5'-GGCACTGTTGTTTCACAAGATGATG-3' |
| hsa- PTEN-R | 5'-TTAGCTGGCAGACCACAAACTGAG-3' |
| hsa- Bcl-xl-F | 5'-AGCTTGGATGGCCACTTACCTG-3' |
| hsa-Bcl-xl-R | 5'-TGCTGCATTGTTCCCATAGAGTTC-3' |
| hsa- Bcl2-F | 5'- TCGCCCTGTGGATGACTGAG-3' |
| hsa-Bcl2-R | 5'-CAGAGTCTTCAGAGACAGCCAGGA-3' |
| hsa- BAX-F | 5'-GCGAGTGTCTCAAGCGCATC-3' |
| hsa-BAX-R | 5'-CCAGTTGAAGTTGCCGTCAGAA-3' |
| hsa-β-actin-F | 5'-TGGCACCCAGCACAATGAA-3' |
| hsa-β-actin-R | 5'-CTAAGTCATAGTCCGCCTAGAAGCA-3' |
